# Prelabour Caesarean Section following IVF/ICSI in Older-Term Nulliparous Women: Too Precious to Push?

**DOI:** 10.1155/2011/362518

**Published:** 2011-11-03

**Authors:** E. Gillet, E. Martens, G. Martens, H. Cammu

**Affiliations:** ^1^Department of Obstetrics & Gynaecology, University Hospital, Vrije Universiteit (VUB), Laarbeeklaan 101, 1090 Brussels, Belgium; ^2^The Flemish Centre for the Study of Perinatal Epidemiology (SPE), Hallepoortlaan 27 B, 1060 Brussels, Belgium

## Abstract

*Objective*. To compare prelabour caesarean section (CS) rates in older nulliparous women with a term singleton baby in cephalic presentation conceiving spontaneously and through IVF/ICSI. When the latter women would ask for CS, how willing are gynaecologists to comply with that request? *Methods*. A population-based retrospective (1995–2009) cohort study, conducted in Northern Belgium. A comparison of 1,866 nulliparous women pregnant after IVF/ICSI and 15,228 controls is made. An anonymous postal questionnaire is sent to all Belgian gynaecologists. *Result*. Both groups are comparable with respect to maternal age, gestational age, and birth weight. Prelabour CS is more often performed in women who conceived through IVF/ICSI compared to those who conceived spontaneously (9.2% versus 6.3%, *P* < 0.001). One in five gynaecologists agrees with the maternal request. *Conclusion*. IVF/ICSI pregnancies in older nulliparous women more often end in a prelabour CS and a substantial number of gynaecologists go along with a nonmedical reason for CS.

## 1. Introduction

Caesarean sections (CSs) now exceed rates thought capable of delivering health benefits to mothers and babies [1]. Indications to perform this major operation have changed over the past decade [2]. Caesarean delivery on maternal request (CDMR) has been added to this list of indications and considered an important contributing factor in the CS rising rate [3–5]. The latter occurs when a woman asks to schedule a purely planned caesarean section on a date mutually convenient for her and her doctor, before the onset of labour, without any medical rationale, and in the absence of any clinical indication. Women's preference can be affected by tokophobia (fear of childbirth), the risk of out-of-hour delivery, the belief that caesareans are safest for the baby, or because vaginal delivery has become stigmatised as archaic and disfiguring [6]. However, in a review of the literature on women's request for caesarean section, it turned out that few women request this procedure in the absence of current or previous obstetric complications [7, 8]. 

A growing number of women achieve their first pregnancy late in life [9–11]. In Flanders, from 1995 until 2010 the percentage of primiparous women being 35 years or older increased from 3.6% to 8.3% (SPE, annual reports). Fertility decreases with age. Consequently, older women will make more use of assisted reproductive technologies (ART). Babies, conceived after ART, of older primiparous women may therefore be considered as “Precious Babies” [12]. 

The aim of this study is to find out whether nulliparous women, aged 35 or more, giving birth after in vitro fertilisation (IVF) or intracytoplasmatic sperm injection (ICSI) are more prone to a prelabour caesarean delivery by comparing them with nulliparous women of the same age who conceived spontaneously. An additional purpose is to evaluate the attitude among Belgian gynaecologists in case of a fictitious primigravid woman of advanced maternal age, pregnant after years of infertility, who asks for a caesarean section. 

## 2. Patients and Methods

The region of Flanders (Northern, Dutch-speaking part of the constitutionally federal state of Belgium) covers 13,521 km^2^ and has about 6.2 million inhabitants. The region has 68 fully equipped maternity units where 99.8% of all births occur. Four of these units are in university hospitals, 15 in other teaching hospitals, and 49 in nonteaching hospitals. Eighteen of the 68 hospitals have an ART unit. Patients from peripheral hospitals are sent to these hospitals, but the policy is that for further followup and delivery, they return to their original gynaecologist. For each newborn of at least 0.5 kg, an official perinatal form must be completed and sent to the Flemisch Centre for Perinatal Epidemiology (SPE, Studiecentrum voor Perinatale Epidemiologie), where all data are controlled by an error detection program and feedback is provided. Perinatal data for this retrospective cohort study is collected from the existing computer files maintained by the SPE. 

During the study period (January 1, 1995–December 31, 2009), pregnancies with the following inclusion criteria are selected: nulliparous women, age 35 years or older, who delivered a live-born singleton infant in cephalic presentation with a gestational age at the time of delivery of 38 weeks or more and without congenital malformations. Pregnancies conceived by IVF/ICSI and pregnancies conceived spontaneously are compared with respect to maternal age, gestational age, and birth weight. Pregnancies conceived after ovulation induction are not included. Rates of spontaneous vaginal and instrumental delivery, prelabour CS and CS following labour, and induction of labour are determined. Neonatal outcome in both groups is compared. 

In 2007, the Belgian College of Physicians for Mother and Newborn tried to determine, through an anonymous questionnaire that was sent to all Belgian gynaecologists, the willingness of Belgian gynaecologists to perform a caesarean section in some particular cases. The first part of the questionnaire included questions about the age and gender, the amount of years of practice as specialist, and the type of hospital and region in which the gynaecologist was practicing. The second part included questions about the personal preference of labour and delivery management in specific fictive case histories. One such case concerned *a primiparous 38 years old and 165 cm tall woman who became pregnant after the 4th IVF attempt, with a singleton baby of 3 kg in cephalic presentation at 39 weeks of gestation. The woman requests a prelabour CS because of fear and because she assumed that it will probably be her only child. *The gynaecologists could either agree or disagree with this demand or they could have no opinion.

## 3. Results

Between 1995 (January 1st) and 2009 (December 31st), 17,094 women are selected. Of this population, 1,866 conceived by IVF and ICSI, while 15,228 conceived spontaneously. Women in both groups are comparable with respect to maternal age, duration of gestation, and birth weight (Table 1). During the study period, the number of older primiparous women who conceived spontaneously increased from 595 in 1995 to 1472 in 2009 (×2.5). The number of women pregnant through IVF/ICSI and having their first baby at advanced maternal age also increased more dramatically from 64 women in 1995 to 224 women in 2009 (×3.5) in the study population. In a period of fifteen years, the amount of so-called “precious babies” more than tripled in Flanders.  Table 2 shows that the labour and delivery characteristics are comparable for both groups, except for prelabour CS. The latter occurred significantly more frequently in those women who got pregnant after IVF/ICSI—relative risk 1.42 (95% confidence interval 1.22–1.64)—compared to spontaneous pregnancies. The neonatal mortality and morbidity was low and comparable in both groups (Table 3). 

**Figure 1:**
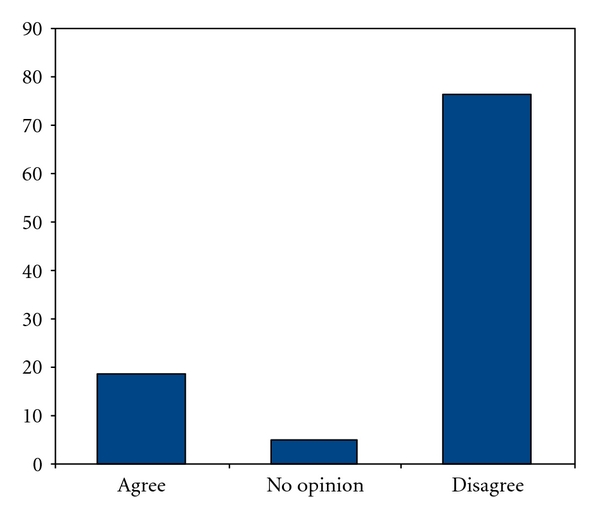
Answers of Belgian gynaecologists concerning a “nulliparous woman of 38 years, pregnant through IVF after 4 attempts and requesting a prelabour caesarean section”.

From a total of 1172 survey forms that reached the Belgian gynaecologist, there was a response rate of 54%, resulting in 632 answers for analysis. The questionnaire reveals that 19% (*N* = 117) gynaecologists would perform a prelabour caesarean section on maternal request (Figure 1). Univariate analysis shows that there is neither a significant difference according to gender nor according to the type of institution to which the gynaecologist belonged (university institution, reference centres, and peripheral hospitals). However, the age of the gynaecologist (*P* = 0.001) and the number of individual annual deliveries (*P* = 0.008) significantly influence the response rate. The gynaecologists of ≥50 years and those performing less than 150 deliveries per year were more in favour to perform a caesarean section in the aforementioned particular case.

## 4. Discussion

This study shows a significant higher rate of prelabour CS in at term women of advanced maternal age (35 years old or more), who become pregnant after IVF/ICSI and have a singleton baby in cephalic presentation, compared to women with the same characteristics who conceive spontaneously. Although the prelabour CS rates are not very high (9.2% versus 6.3%), the data suggest that there is a lower threshold for performing a prelabour caesarean section when the pregnancy is artificially conceived. Once in labour, however, no significant differences in CS rates are seen between both groups. These objective data corroborate to some degree with the subjective data that nearly one in five Belgian gynaecologists is willing to perform a CS on maternal request to deliver a “precious baby”. 

Many studies indicate that rates of caesarean section are significantly higher after assisted than after natural conception [13–16]. This practice has previously been described as the “precious baby” effect [12]. Based on knowledge that the pregnancy is the result of ART, gynaecologists may modify their practice to deliver by caesarean section [12]. A systematic review of 25 studies, published between 1985 and 2002, of singleton pregnancies after ART and matched controls, shows that ART pregnancies more often—RR of 1.54 (95% CI 1.44–1.66)—ended in a caesarean section [17]. Australian IVF term women, with singleton babies, underwent significantly more frequently (43.8%) a caesarean section compared to controls (27.8%), OR 2.02 (95% CI 1.95–2.10) [18]. Twin pregnancies, conceived with the aid of ART, are also at increased risk to be delivered by caesarean section, compared to spontaneously conceived twin pregnancies [17, 19]. 

A fairly robust literature shows that ART pregnancies result more frequently in preterm delivery and a low-birth-weight infant [20, 21]. In order to avoid the preterm birth bias, only at term (38 weeks or more gestation) singleton pregnancies in cephalic presentation of nulliparous women of advanced maternal age (>35 years) were included. IVF/ICSI and spontaneous conceived pregnancies were comparable with respect to duration of gestation and birth weight.

It is unclear in this study whether the impetus for this higher rate of prelabour caesarean delivery is the result of a maternal request, a defensive attitude by the physician, or of a consensus between the physician and the pregnant woman. Maternal request may play a role. Women may perceive caesarean delivery to be safer for their babies than vaginal birth [22]. Another possibility for the higher incidence of CS is the fear of the gynaecologist about the outcome of a pregnancy that was difficult to conceive. From 40% to 54% of physicians approve of a woman's right to request and obtain a caesarean section without medical indication (USA, Denmark) [3, 23]. This is substantially higher than the 19% Belgian gynaecologists that, according to the questionnaire, agreed to perform an CS on maternal request. In the USA, an important determinant for performing a prelabour caesarean section for no medical reasons is when the pregnant woman belongs to a high socioeconomic level [3]. A recent study found the opposite, namely, that the frequency of having a prelabour CS level is inversely related to the level of maternal education [22].

An infertility treatment is stressful for the affected couples. Women getting pregnant for the first time at advanced age are even more under pressure. They are more at risk for complications, and they feel rushed into pregnancy if they want to have children before the window of opportunity closes. It is for sure that IVF/ICSI is not an easy way to conceive: it is expensive, success is not assured, and the process itself entails daily injections of hormones, painful procedures, and a great expenditure in time and energy. All these stressful factors could play a role in the decision of mother and doctor to plan a prelabour caesarean section and schedule the delivery on a time convenient for both of them. In many countries governments lack funding for fertility treatment and the financial impact of paying for these treatments can be too much to bear for some families. A reason why the prelabour caesarean section rates for IVF/ICSI pregnancies in Flanders are quite low, compared to other countries [14, 18], may be explained by the high level of reimbursement for those expensive treatments. Patients who fall under the Belgian national health service system are reimbursed for the laboratory costs of fertility treatments and for the larger part of the cost of stimulation medication [24]. Women can receive up to six IVF cycles free of charge. The cost reimbursed per IVF/ICSI cycle in Belgium is €2426 (2004), but the patients have to pay only €200 [25]. 

Until evidence supports prelabour caesarean section as a birth option that optimizes outcomes for mothers and their infants, obstetricians should continue to support evidenced-based decision making that includes advocacy for vaginal delivery as the optimal mode of birth. Fever, infection, pneumonia, and thromboembolic events are increased with caesarean delivery [6]. Besides, prelabour caesarean deliveries that are performed before 39 weeks of gestation are associated with adverse neonatal outcomes such as mechanical ventilation, newborn sepsis, hypoglycaemia, and admission to the neonatal ICU [26].

## 5. Conclusion

 In Northern Belgium (Flanders), pregnancies after IVF/ICSI are more prone to end in a prelabour caesarean section, putting an additional burden on the economic expenditure for obstetric care in assisted pregnancies. One in five obstetricians is willing to do a caesarean section on demand. No matter how stressful an IVF/ICSI pregnancy can be, indication for caesarean section should be evidence based.

##  Conflict of Interests

There was no conflict of interests nor a financial disclosure for any of the authors.

##  Authors Contributions

E. Gillet wrote the manuscript; E. Martens collected the data; G. Martens did the statistical analysis; H. Cammu designed the study and revised the manuscript. 

## Figures and Tables

**Table 1:** Patient characteristics.

	IVF and ICSI pregnancies (*n* = 1,866)	Spontaneous pregnancies (*n* = 15,228)	*P* value
Maternal age (y ± SD)	37.8 ± 2.4	37.3 ± 2.0	NS
Gestational age (wk ± SD)	39.5 ± 1.0	39.6 ± 1.0	NS
Birth weight (g ± SD)	3,362 ± 449.5	3,363 ± 449.3	NS

IVF: in vitro fertilisation, ICSI: intracytoplasmatic sperm injection, y: years, g: grams, SD: standard deviation, NS: not significant.

**Table 2:** Labour characteristics.

	IVF and ICSI pregnancies (*n* = 1,866)	Spontaneous pregnancies (*n* = 15,228)	*P* value	RR (95% CI)
Spontaneous delivery	969 (51.9%)	7,996 (52.5%)	0.65 (NS)	0.98 (0.90–1.07)
Instrumental delivery	424 (22.7%)	3,759 (24.7%)	0.067 (NS)	0.91 (0.82–1.01)
Prelabour CS	171 (9.2%)	966 (6.3%)	<0.001	1.42 (1.22–1.64)
CS in labour	302 (16.2%)	2,506 (16.5%)	0.8 (NS)	0.98 (0.87–1.10)
Induction of labour	661 (35.4%)	5051 (33.2%)	0.051 (NS)	1.09 (1.00–1.20)

IVF: in vitro fertilisation, ICSI: intracytoplasmatic sperm injection, CS: caesarean section, RR: relative risk, CI: confidence interval, NS: not significant.

**Table 3:** Neonatal outcome.

	IVF and ICSI pregnancies (*n* = 1,866)	Spontaneous pregnancies (*n* = 15,228)	*P* value
Early neonatal death	2 (0.11%)	9 (0.06%)	0.77 (NS)
Transfer to NICU	62 (3.3%)	394 (2.6%)	0.074 (NS)
Intubation	13 (0.7%)	68 (0.4%)	0.19 (NS)
Convulsions	3 (0.16%)	19 (0.12%)	0.95 (NS)

IVF: in vitro fertilisation, ICSI: intracytoplasmatic sperm injection, NICU: neonatal intensive care unit, NS: not significant.
